# Metal ion stimulation-related gene signatures correlate with clinical and immunologic characteristics of glioma

**DOI:** 10.1016/j.heliyon.2024.e27189

**Published:** 2024-02-28

**Authors:** Chengzhi Jiang, Binbin Zhang, Wenjuan Jiang, Pengtao Liu, Yujia Kong, Jianhua Zhang, Wenjie Teng

**Affiliations:** a Shandong Second Medical University, Weifang, Shandong, 261053, People's Republic of China; b Qingdao Municipal Hospital (Group), Qingdao, Shandong, 266000, People's Republic of China; c Jining Medical University, Jining, Shandong, 272067, People's Republic of China

**Keywords:** Supervised clustering, Metal ion stimulation glioma

## Abstract

**Background:**

Environmental factors serve as one of the important pathogenic factors for gliomas. Yet people focus only on the effect of electromagnetic radiation on its pathogenicity, while metals in the environment are neglected. This study aimed to investigate the relationship between metal ion stimulation and the clinical characteristics and immune status of GM patients.

**Methods:**

Firstly, mRNA expression profiles of GM patients and normal subjects were obtained from Chinese GM Genome Atlas (CGGA) and Gene Expression Omnibus (GEO) to identify differentially expressed metal ion stimulation-related genes(DEMISGs). Secondly, two molecular subtypes were identified and validated based on these DEMISGs using consensus clustering. Diagnostic and prognostic models for GM were constructed after screening these features based on machine learning. Finally, supervised classification and unsupervised clustering were combined to classify and predict the grade of GM based on SHAP values.

**Results:**

GM patients are divided into two different response states to metal ion stimulation, M1 and M2, which are related to the grade and IDH status of the GM. Six genes with diagnostic value were obtained: SLC30A3, CRHBP, SYT13, DLG2, CDK1, and WNT5A. The AUC in the external validation set was higher than 0.90. The SHAP value improves the performance of classification prediction.

**Conclusion:**

The gene features associated with metal ion stimulation are related to the clinical and immune characteristics of transgenic patients. XGboost/LightGBM Kmeans has a higher classification prediction accuracy in predicting glioma grades compared to using purely supervised classification techniques.

## 1 Introduction

GM is a collective term originating from glial cells and neuronal cells of the nervous system, and is the most common malignant tumor in the skull, accounting for 40%–50% of intracranial tumors. GM can be classified into astrocytomas, glioblastomas(GBM), oligodendroglioma, and other types according to the cell types. The incidence of GM is predominantly male [1], the diagnosis is complex, and the incidence rate is increasing year by year, with an annual growth rate of about 1.2%, and the number of deaths reaches 30,000 per year [2]. The symptoms of GM are not obvious. It is difficult to completely resect. GM is less sensitive to radiotherapy and chemotherapy. It is very easy to recur. GM is one of the tumors with the worst prognosis among systemic tumors. Currently, it seems that IDH1, P53, and GFAP are biomarkers for GM diagnosis [[2], [3], [4]], and IDH mutation and CDKN2A deletion are biomarkers for predicting the prognosis of GM [5]. However, the current biomarkers are far from meeting the clinical needs.

GM is caused by the interaction of innate genetic risk factors and environmental carcinogenic factors. In addition to internal genetic factors, more and more studies have been conducted on environmental factors as one of the important factors in the occurrence and development of GM [[6], [7], [8], [9], [10]]. Radiation as a component of environmental factors has been widely emphasized in studies of transgenic pathogenicity. However, metals in the environment have also been overlooked as possible pathogenic factors [[11], [12], [13]]. Metals entering the human body exist in the form of ions. By forming biocompatibility with biological ligands such as proteins and nucleic acids, metal proteins and metalloenzymes are formed. Plays important biochemical and physiological roles in the process of life [[14], [15], [16], [17]]. However, if they are not chelated, they can also catalyze destructive metal substitution reactions or non-specific redox reactions. This can lead to changes in the state or activity (in terms of motility, secretion, enzyme production, and gene expression) of a cell or organism stimulated by metal ions. In recent years, metal ion stimulation and neurodegenerative diseases have been extensively studied [[18], [19], [20], [21], [22]]. It has also been confirmed that neurodegenerative diseases and transgenesis share striking similarities and overlaps in mechanisms and pathways, and are in fact two sides of the same coin [23]. Metal ion stimulation is also closely related to the human immune system. This suggests a potential association between metal ion stimulation and GM. In light of this, the relationship between metal ion stimulation and GM is unexplored and deserves in-depth study.

In recent years, the field of machine learning has grown rapidly. Classification or regression tasks are inevitably encountered in the study of GM pairs, and efficiency in handling these tasks is very important. To improve the efficiency of machine learning models in these tasks, choosing reasonable features is one aspect, and tuning the model is another. The most important thing is to optimize the model, algorithmic computation, or workflow to improve the accuracy of the classification task and reduce the error in the regression task. The presence of SHAP values makes the so-called “black box model” more interpretable. For each predicted sample, the model generates a predicted value, and each feature of each sample has a corresponding SHAP value, which represents the influence of that feature on the prediction [24,25]. The process of calculating SHAP can be regarded as the process of feature amplification. And combining it with unsupervised clustering can optimize the effect of supervised clustering. Then the confusion matrix is constructed by grouping the sample labels with the clustering results, and the precision and other metrics can be calculated for classification. The advantages of supervised clustering based on SHAP values have been demonstrated for a long time [26], and this study improves on it to increase the accuracy of classification prediction.

Here, we take metal ion stimulation as the starting point of our study and focus on its relationship with the clinical and immune characteristics of GM. The research goal is to improve the diagnosis, treatment, and prognosis of GM patients and to lay the foundation for the prevention of GM. Meanwhile, in the course of the study, an attempt was made to connect both supervised classification and unsupervised clustering using SHAP. This allows for greater efficiency in the classification prediction task and provides new options when choosing a model for dealing with the classification task.

## 2 Materials and methods

### 2.1 Sample source

Transcriptome sequencing datasets GSE4290 (n = 180) and GSE50161 (n = 130) for the GM and normal groups were obtained from the GEO database; transcriptome sequencing datasets mRNAseq_693 (n = 693) and mRNAseq_325 (n = 325) for GM patients were obtained from the CGGA database. The set of characterized genes associated with stimulation in response to metal ions was obtained from the MSigdb database and the Reactome database [27,28], and the total number of characterized genes was 368.

### 2.2 Differential expression gene analysis

DEG analyses were performed using the “limma” package for the R(4.1.0) language [29,30]. The first time was to analyze the difference between the GSE50161 GM group and the normal group; the second time was to analyze the difference between the different subgroups of metal ion stimulation response. The cutoff value of logFC during these analyses was 2, and the truncated value of the corrected FDR value q-value is 0.05. After DEG analysis, GO and GSEA functions and pathway enrichment were analyzed [31,32].

### 2.3 Machine learning algorithms

The study utilized machine learning algorithms such as XGboost, LightGBM [32], and logistic regression [[33], [34], [35], [36]]. These algorithms enabled effective processing of large-scale glioma transcriptomic data and facilitated prediction of the malignancy and prognosis of gliomas through methods such as pattern recognition, classification, and regression. Based on this, the study could assist researchers in identifying key genes and molecular markers related to the occurrence and progression of gliomas, offering new insights for the early diagnosis and treatment of the disease. Before constructing the machine learning models, we adjusted their parameters. The XGboost model was tuned using grid search with 5-fold cross-validation, while the LightGBM model was tuned using Bayesian optimization with 5-fold cross-validation. During this process, the ‘caret’ package in R Studio was used to implement grid search for cross-validation to find the optimal model parameters. The “trainControl” function provided nearly 10 different cross-validation methods, including “cv”, “repeatedcv”, “LOOCV”, “LGOCV”, and others. The 'tidymodels' package implemented Bayesian tuning for cross-validation, with the “vfold_cv” function offering two cross-validation methods: “cv” and “repeatedcv”. The parameters “max_depth”, “eta”, “gamma”, “colsample_bytree”, “min_child_weight”, and “subsample” of the XGboost model were adjusted. The parameters “trees”, “min_n", “tree_depth”, " learn_rate” parameters were tuned. In addition, we used two feature selection methods: recursive feature elimination [37] and SHAP value method. The recursive feature elimination algorithm is a greedy algorithm, the main idea is to iteratively construct the model (XGboost model) and then select the best (or worst) features, select the selected features, and then repeat the process on the remaining features until all the features have been traversed. The SHAP (SHapley Additive exPlanations) algorithm is a method that aims to improve the interpretability of black-box machine learning models by explaining each prediction result. This method is based on the concept of Shapley values from cooperative game theory and is used to address the issue of interactions among multiple features, specifically how to explain a prediction result when there are interactions among multiple features.

### 2.4 Subgroup identification

We used consensus clustering to identify potential subtypes of GM stimulated by metal ions. This method was implemented using the ConsensusClusterPlus function in the “ConsensusClusterPlus” package [38]. In this function, the parameters were set as follows: maxK = 6, reps = 1000, pItem = 0.8, pFeature = 1, clusterAlg = "km”, and distance = "euclidean”. The number of clusters was determined by integrating information from CDF plots, Cluster-Consensus Plots, and cluster-consensus values. After determining the optimal number of clusters, we used tSNE to confirm the correctness of the grouping. The tSNE dimensionality reduction results for each sample were summed to obtain an individual's score for the response to metal ion stimulation.

### 2.5 Estimating the infiltration of immune cells

The quanTIseq algorithm was used to assess the percentage of immune cells in each sample [39]. quanTIseq was used to quantify tumor immune status based on human RNA-seq data, quantifying the proportion of the 10 different immune cell types present in the sample as well as the proportion of other uncharacterized cells by back-convolution.

### 2.6 Building a prognostic model

Using Kaplan-Meier survival analysis, univariate and multivariate cox regression we designed a risk model containing five metal ion stimulation-related genes that are strongly associated with GM prognosis. Based on the expression of each gene in each sample in order to calculate the risk score. Based on the median risk score, GM patients were categorized into high and low risk groups. To demonstrate the reliability of the risk model. Datasets mRNAseq_325 and mRNAseq_693 were used for training and validation, respectively. In addition, we examined the relationship between risk scores and clinical factors.

### 2.7 Construction of a glioma grade prediction model

In the classification of gliomas, the World Health Organization (WHO) classifies them into grades I-IV, with grades I and II being low-grade gliomas, and grades III and IV being high-grade gliomas [40]. Therefore, the tumor grade of glioma patients is categorized as either high-grade glioma or low-grade glioma as the outcome variable. For the prediction of glioma grade, we constructed classification prediction models using XGBoost, LightGBM, XGBoost-Kmeans, and LightGBM-Kmeans. The XGBoost-Kmeans model is a new model built on the basis of the XGBoost model. The XGBoost model was established using the training set, and SHAP was then used to explain the external validation set and obtain the SHAP values for each individual in the external validation set. These SHAP values were used as input to construct the K-means model, with the number of clusters (k) chosen as 2 to maintain consistency with the number of glioma grades. The clustering results and the original labels of the samples were used to construct a confusion matrix to evaluate XGBoost-Kmeans models. The construction process for the LightGBM model and the LightGBM-Kmeans model is identical to the construction process for the XGBoost and XGBoost-Kmeans models.

### 2.8 Statistical analysis

The difference between the two samples was compared using the *t*-test of the two samples. Pearson correlation coefficient was used to analyze the correlation between two consecutive data. The difference between the two groups of data was compared usingthe chi-squaree test. The correlation analysis between the two groups of data used Cramer's V correlation coefficient.

## 3 Results

### 3.1 Landscape of mutation profiles in GM samples

**Fig. 1:**
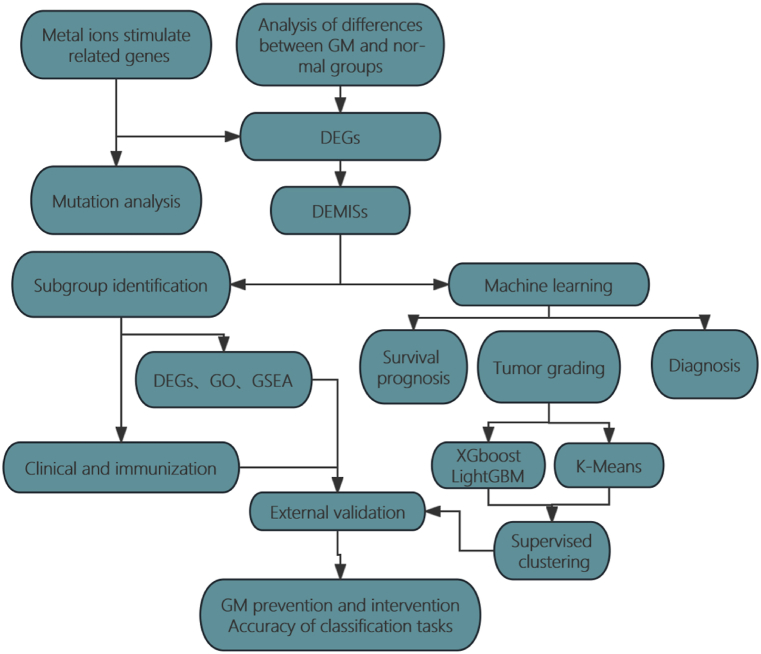
The workflow of this study.

**Fig. 2:**
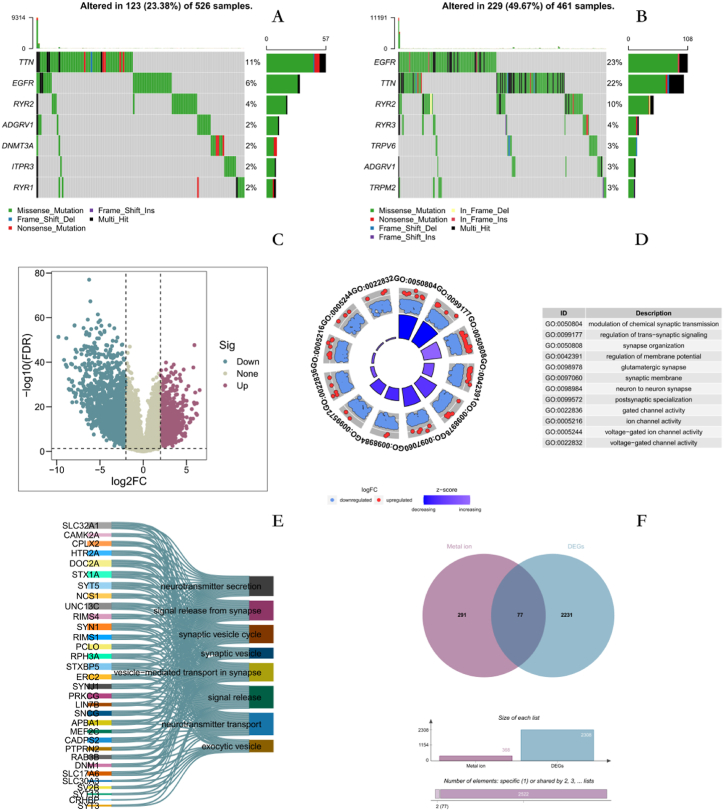
Differentially expressed gene analysis. A Mutational status of metal ion stimulation-related genes in LGG. B Mutational status of metal ion stimulation-related genes in GBM. C Differentially expressed genes in GM patients and normal group. D GO enrichment analysis results. E GSEA enrichment analysis results. F Access to DEMISGs (In the upper part of F, the light pink set represents the gene set of metal ion stimulation characteristics, while the light blue set represents the differential gene set between the GM group and the normal group. The overlapping part of these two gene sets is differentially expressed metal ion stimulation related genes (DEMISGs) The middle section of F and the upper part of the graph are basically consistent, representing the number of different gene sets. The lower part of F represents the overlapping and non overlapping parts of two gene sets).

The research flow of the entire analysis is shown in (Fig. 1). Mutation data of 526 Low-grade GM(LGG) patients and 461 GBM patients were obtained from the TCGA database to analyze the mutation status of metal ion stimulation-related signature genes in both diseases. The results showed that TNN, EFGR, and RYR2 in LGG ranked the top three in terms of mutation frequency, with the largest percentage of mistranslated mutations in different mutation categories (Fig. 2A). EFGR, TNN, and RYR2 in GBM ranked the top three, also with the largest percentage of mistranslated mutations in different mutation categories (Fig. 2B). Overall, the mutation frequency of metal ion stimulation-related characterized genes was much higher in GBM than in LGG.

### 3.2 Identification of metal ion stimulation-associated DEGs in GM

Differences in gene expression between the GM and normal groups were analyzed in dataset GSE50161, and 2308 differentially expressed genes(DEGs) were obtained, of which 911 were up-regulated and 1397 were down-regulated (Fig. 2C). These genes were analyzed by GO(Gene Ontology) and GSEA(Gene Set Enrichment Analysis) enrichment, and the results showed that synapse organization, glutamatergic synapse, and neurotransmitter secretion were enriched (Fig. 2D and E). Seventy-seven overlapping genes were obtained by taking the intersection of the above differential genes with the metal ion stimulation-associated signature genes (Fig. 2F). These genes are called DEMISGs in GM patients.

### 3.3 Metal ion stimulation-related genes identify different metal ion stimulation response states

**Fig. 3:**
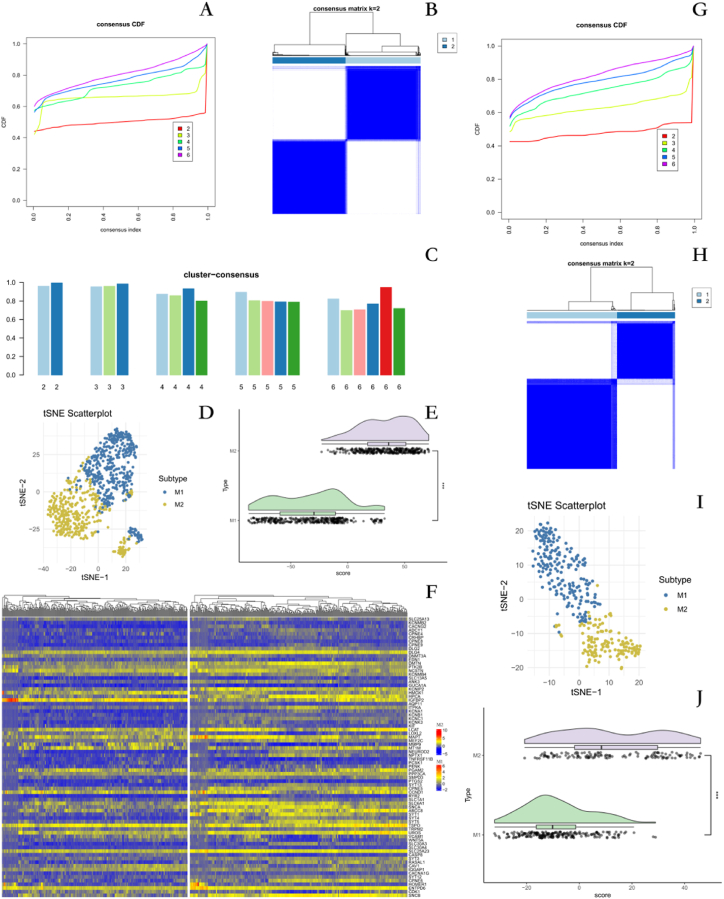
Identifying subtypes of GM patients in response to metal ion stimulation **A** Consistency index and CDF graph under different cluster numbers in training set **B** Consistent cluster matrix in training set k is 2 **C** Cluster-consensus value graph under different cluster numbers in training set **D** The correct grouping of the two subtypes was determined by tSNE analysis in the training set. **E** The training focused on quantifying the response of different subgroups of GM patients to metal ion stimulation. **F** Differential expression of DEMISGs in the two subgroups. **G** Consistent cluster matrix in test set k is 2. **H** Consistency index and CDF graph under different cluster numbers in test set. **I** The correct grouping of the two subtypes was determined by tSNE analysis in the test set. **J** The test focused on quantifying the response of different subgroups of GM patients to metal ion stimulation.

Consensus cluster analysis was performed based on 76 DEMISGs (1 gene deletion). The results of the CDF plot indicate that when k = 2, the CDF reaches an approximate maximum value(Fig. 3A). The matrix heatmap shows that k = 2 is a very pure clustering(Fig. 3B). The Cluster Consensus Plot indicates that when k = 2, the stability of the cluster reaches its highest level(Fig. 3C). The tSNE analysis showed no significant overlap between the two clusters(Fig. 3D). Based on the above results, k = 2 exhibits excellent clustering stability, with the highest intra-group correlation and the lowest inter-group correlation. Therefore, GM patients were categorized into two subtypes: clusters M1 and M2. The score of each individual's response to metal ion stimulation was calculated using tSNE, which demonstrated a higher score for M2 than for the M1 group(Fig. 3E). The heatmap demonstrated that the expression of the 76 DEMISGs differed between the two subtypes(Fig. 3F). To further validate the rationality of identifying two different states of response to metal ion stimulation based on 76 DEMISGs, we again used consensus clustering to categorize the GM patients in the mRNAseq_325 dataset into different subgroups(Fig. 3G-I). The metal ion stimulation response score of M2 group is higher than that of M1 group(Fig. 3J).

### 3.4 Responses to stimulation by metal ions in GM show different clinical and immunologic features

**Fig. 4:**
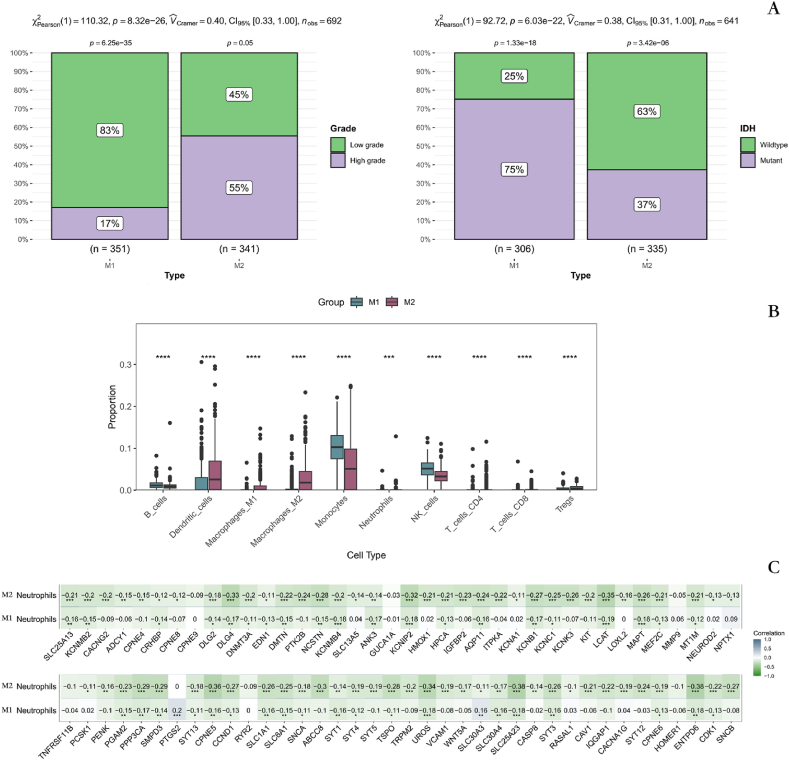
The clinical and immunological relationship between different subtypes and GM patients **A** The relationship between different subtypes and GM grading and IDH status. **B** Differences in the percentage of immune cells of different subtypes. **C** Relationship between immune cell percentage and DEMISGs expression in M2 group. (One * indicates a *P*-value less than 0.05, two * indicates a *P*-value less than 0.01, and three * indicates a *P*-value less than 0.001).

We investigated the relationship between two different metal ion stimulation response states and the clinical features of GM. There was a preponderance of high-grade GM patients in the M2 group and a preponderance of wild-type in the mutation state (Fig. 4A). In addition, we quantified the percentage of 10 immune cells accounted for by both subtypes using the quanTIseq algorithm. Dendritic cells, Macrophages M1, Macrophages M2, Tregs, and CD8 T cells were more prevalent in the M2 subtype compared to the M1 subtype, which had higher proportions of B cells, Monocytes, and NK cells than the M2 subtype (Fig. 4B). We further analyzed the correlation between the proportions of the 10 immune cells and the expression of DEMISGs. The results showed that the expression of most DEMISGs in the M1 subtype showed a strong positive correlation with B cells, and the expression of most DEMISGs in the M2 subtype showed a strong positive correlation with M1 macrophages, M2 macrophages, and Tregs. The correlation between the expression of DEMISGs and Neutrophils in the M1 subtype was much weaker than that in the M2 subtype (Fig. 4C).

**Fig. 5:**
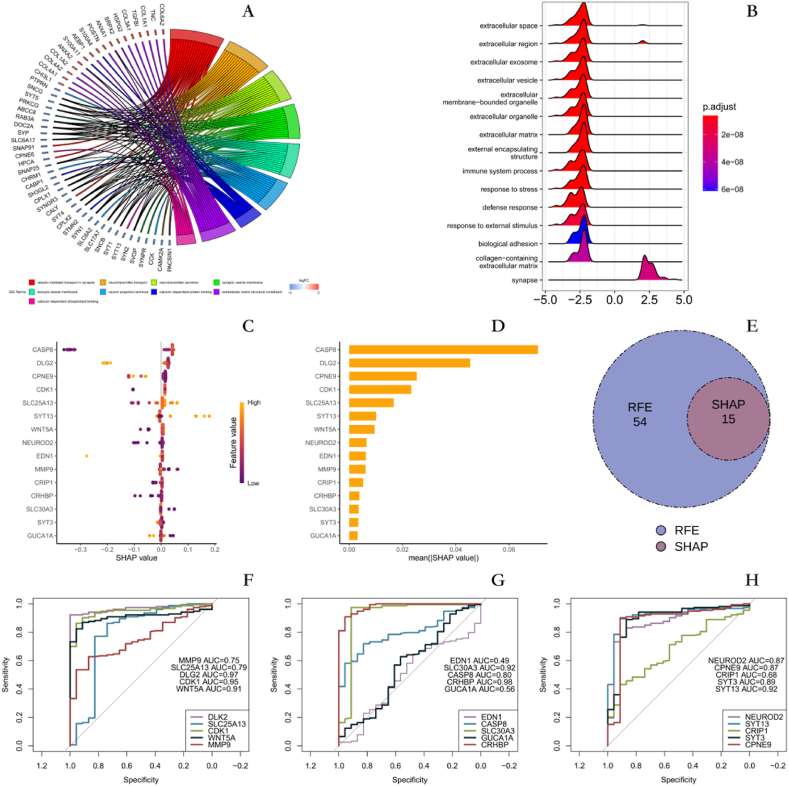
Identifying new diagnostic biomarkers for GM A GO enrichment analysis of differentially expressed genes between M1 and M2 groups. B GSEA enrichment analysis of differentially expressed genes between M1 and M2 groups. C The contribution of each variable to the model prediction D Selection of key features based on SHAP values. E Overlapping key features selected by recursive feature elimination and SHAP value method. F–H ROC analysis to evaluate the diagnostic value of biomarkers.

We also performed DEG analysis of two different metal ion stimulation response states and identified 130 up-regulated genes and 87 down-regulated genes. GO analysis showed that: neurotransmitter transport, vesicle-mediated translocation in the synapse, neurotransmitter secretion, calcium-dependent protein binding were enriched (Fig. 5A). GSEA enrichment analysis showed that: synapse, response to external stimulus, immune system process were enriched (Fig. 5B).

### 3.5 Identifying GM diagnostic biomarkers related to metal ion stimulation features

A search for new biomarkers that could improve transgenic diagnostics based on 77 DEMISG. Regression models were built using XGboost, and feature selection was performed using recursive feature elimination (RFE) and calculation of SHAP values. Logistic regression was used to assess the diagnostic value of the screened features. Recursive feature elimination based on 5-fold cross-validation was performed on the established XGboost regression model. Sixty-nine features were obtained after 385 iterations. DLG4, KCNA1, LCAT, PPP3CA, SLC1A1, SNCA, SLC30A4 and SLC25A23 were eliminated. SHAP values were then calculated for the established XGboost regression model and 15 characterized genes were identified based on the SHAP values. These included DLG2, SLC25A13, CDK1, WNT5A, MMP9, EDN1, CASP8, SLC30A3, GUCA1A, CRHBP, NEUROD2, SYT13, CRIP1, SYT3 and CPNE9(Fig. 5C and D). A total of 15 overlapping features were obtained by both methods. This is consistent with the features obtained based on SHAP values (Fig. 5E).

Logistic regression models were constructed based on the 15 feature genes using 70% of the data from GSE50161 as a training set. The diagnostic models constructed for each feature were evaluated using ROC analysis in the external validation set GSE4290. The results showed that the AUC of SLC30A3, CRHBP, SYT13, DLG2, CDK1, and WNT5A in the external validation set was higher than 0.90 (Fig. 5F–H).

### 3.6 Construction and analysis of a prognostic model related to metal ion stimulation

**Fig. 6:**
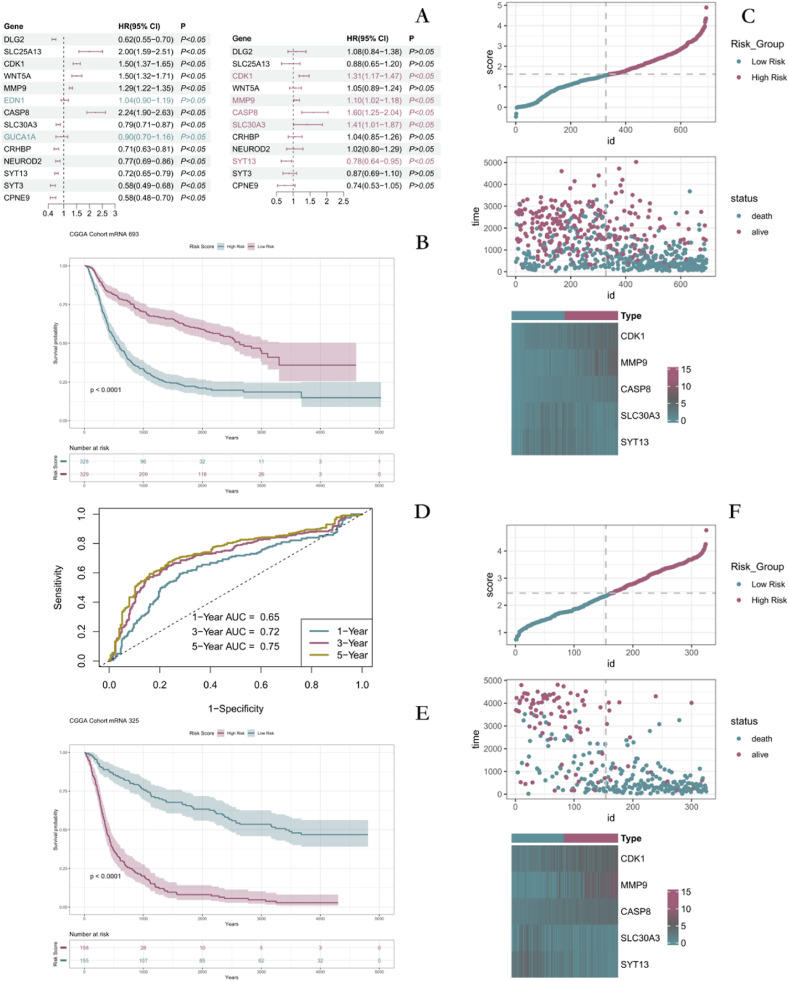
Survival analysis **A** Results of single-factor and multifactor Cox regression. **B**–**C** Survival analysis results. **D** Predicting 1-, 3-, and 5-Year Survival in GM Patients. **E**-**F** External validation of survival analysis results.

**Fig. 7:**
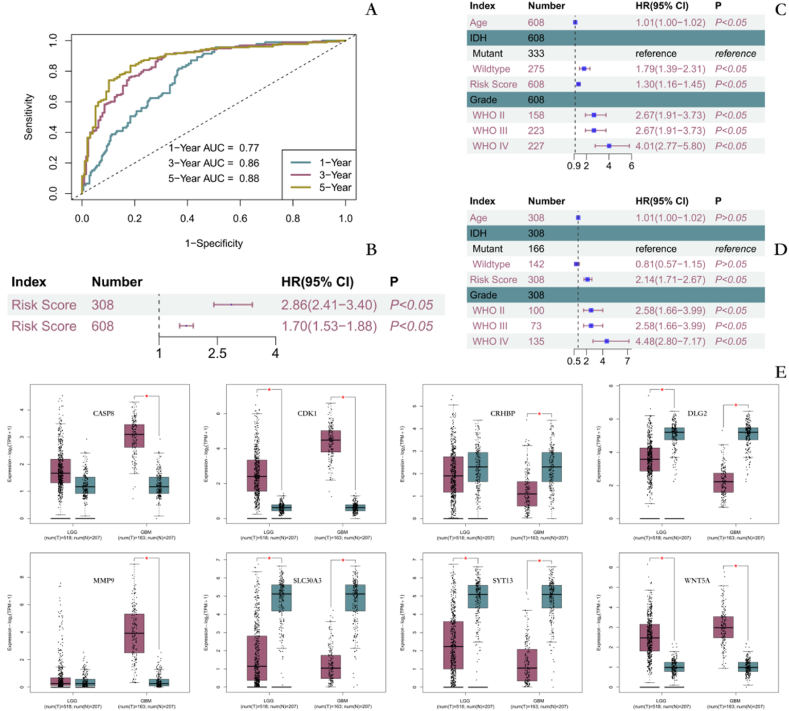
Constructing prognostic models and validating the expression of relevant genes **A** Validation for predicting survival in GM patients. **B**-**D** Analysis and validation of risk score as an independent risk factor for prognosis in GM patients. **E** Validation of gene expression with diagnostic and prognostic value. (The red asterisk represents a p-value less than 0.01).

Twelve DEMISGs associated with survival were identified using univariate Cox regression in the dataset mRNAseq_693. Multivariate Cox regression was then used to select statistically significant 5 genes and to construct a prognostic model for patients with GM after calculating the regression coefficients (Fig. 6A). TThe risk score was calculated as 0.2695063*CDK1+0.0924100*MMP9+0.4679576*CASP8+0.34006645*SLC30A3-0.2472611*SYT13. The patients were categorized into high-risk and low-risk groups based on the median risk scores. The Kaplan-Meier curves showed that the patients in the high-risk group had a significantly shorter survival time than those in the low-risk group. Prognostic gene expression also differed between the two groups (Fig. 6B and C). Time-dependent ROC analysis showed that risk scores could accurately predict OS in cancer patients (all AUCs were above 0.75) (Fig. 6D). The above results were validated in mRNAseq_325 (Fig. 6E and F, Fig. 7A).

To demonstrate that risk score was an independent prognostic factor for GM, univariate Cox regression was first performed in both datasets. The results indicated that risk score was a risk prognostic factor for GM patients (Fig. 7B). Multivariate Cox regression was performed by combining age, IDH, and grading of GM patients. The results still indicated that risk score was a risk prognostic factor for GM patients (Fig. 7C and D). Thus, risk score was identified as an independent prognostic factor for GM patients. We validated the expression of prognostic and diagnostic genes by the GEPIA2 platform (Fig. 7E).

### 3.7 Construction and stability verification of glioma classification and prediction model

80% of mRNAseq_693 was used as the training set, and mRNAseq_325 was used as the external validation set. A classification model based on XGBoost was established in the training set using 5-fold cross-validation and grid search optimization. The model was then used to make predictions in the external validation set, and a confusion matrix was constructed based on the prediction results. Subsequently, SHAP values were used to explain the external dataset, and a K-means clustering (k = 2) was performed on the explained new data to build the XGBoost-Kmeans model. The clustering results were combined with the existing grouping labels to construct a confusion matrix. The process of building the LightGBM model and the LightGBM-Kmeans model, based on Bayesian adjusted parameters, was consistent with the process of building the XGBoost and XGBoost-Kmeans models.

**Fig. 8:**
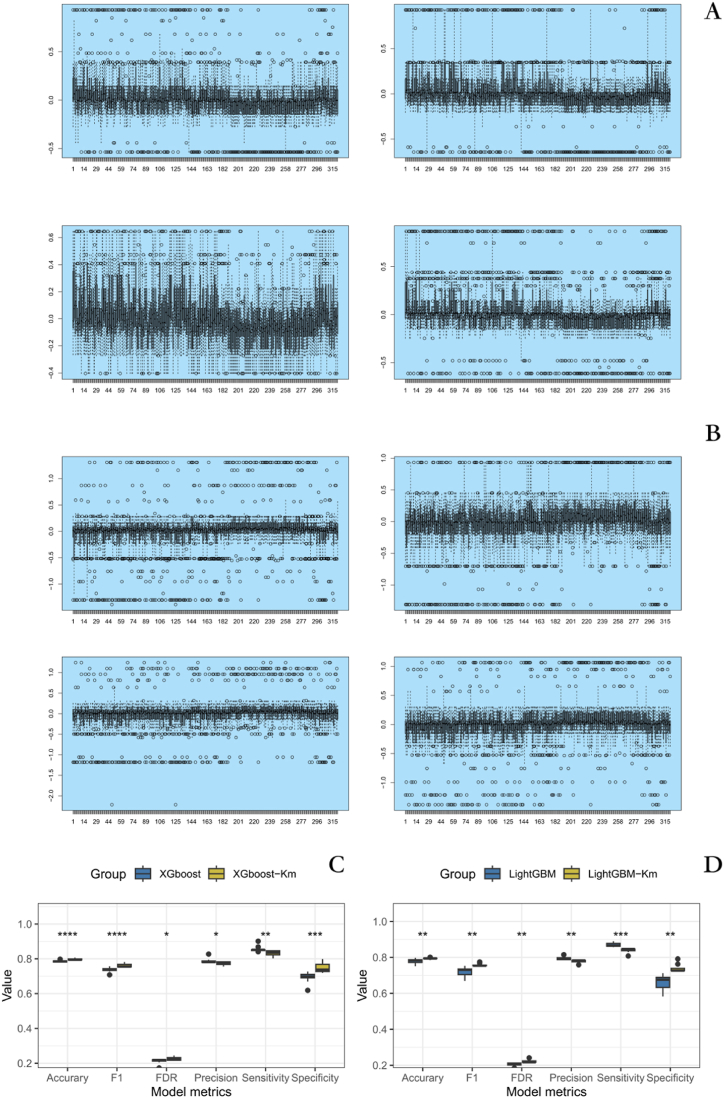
The performance of four classification prediction models under different splitting schemes **A** The performance of individual SHAP values in an externally validated set under the LightGBM model. **B** The performance of individual SHAP values in an externally validated set under the XGboost model. **C** Comparison of classification prediction performance of XGboost and XGboost-Kmeans models under different segmentation schemes **D** Comparison of classification prediction performance of LightGBM and LightGBM-Kmeans models under different segmentation schemes. ((One * indicates a *P*-value less than 0.05, two * indicates a *P*-value less than 0.01, and three * indicates a *P*-value less than 0.001)).

We employed repeated cross-validation with ten different splitting schemes to obtain a more stable GM-level prediction model. We found that when using SHAP to explain the XGboost and LightGBM models on the external validation set, the SHAP values for each individual did not exhibit significant fluctuations(Fig. 8A and B). Due to the large sample size of the external validation set and the use of ten different splitting schemes, we only presented the variations in SHAP values for individuals under four of the splitting schemes to provide a clear representation of the results. Through repeated cross-validation of 10 different splitting schemes, we first compared the predictive differences between the XGboost model and XGboost Kmeans, as well as the LightGBM model and LightGBM Kmeans, using paired t-tests. The results showed significant differences in accuracy, sensitivity, specificity, precision, FDR, and F1 scores between the two groups of models (Fig. 8C and D). Secondly, we will compare the average values of each indicator under repeated cross-validation of 10 different splitting schemes as the final result. We found that the XGBoost Kmeans model outperformed 4 out of these 6 metrics. But for the other two indicators, they performed the worst among these four models (Table 1). This suggests that there seems to be a clear trade-off between these indicators.

**Table 1:** Comparison of classification prediction performance of four models.

	XGBoost	XGBoost-Kmeans	LightGBM	LightGBM-Kmeans
Accuracy	0.79	0.79	0.78	0.79
Sensitivity	0.69	0.75	0.66	0.74
Specificity	0.86	0.83	0.87	0.84
Precision	0.78	0.77	0.80	0.78
FDR	0.21	0.23	0.21	0.22
F1 score	0.74	0.76	0.72	0.76

## 4 Discussion

GM is a malignant tumor with a very poor prognosis due to a combination of innate genetics and acquired environment. Electromagnetic radiation has been widely mentioned in past studies of environmental factors associated with GM. However, a large number of current studies indirectly or directly imply that metals in the environment are closely related to GM [[41], [42], [43], [44]]. Gene expression is altered in humans when stimulated by metal ions, but the relationship between such changes and the grade of GM, IDH status, and immune profile is not clear, and the exact role of metal ion stimulation in GM and the underlying mechanisms are also poorly understood. Given this, we investigated the relationship between metal ion stimulation-related genes and clinical and immune features of GM, improving the diagnosis, treatment, and tumor grading of GM. Furthermore, we found that SHAP plays a crucial role in the process of building the optimal model for predicting the grade of GM.

We first analyzed the mutation status of metal ion stimulation-related genes in LGG and GBM samples. Metal ion stimulation genes were more frequently mutated in GBM patients compared to LGG patients. This suggests that metal ion stimulation is associated with more malignant GM. Then 77 DEMISGs were identified between GM patients and the normal group and analyzed by GO, GSEA enrichment. We found that these genes were mainly enriched in the regulation of chemical synaptic transmission, voltage-gated ion channel activity, and glutamatergic synapses. It has been shown that glutamatergic synapses are present between GM cells and neurons, and that glutamatergic glial synapses drive brain tumor progression by affecting calcium ion signaling and stimulating GM invasion and growth [45]. Voltage-gated ion channel activity has also been shown to influence GM migration and invasion [46]. Chemical synaptic transmission promotes GM development, and the takeover of brain neuronal networks by cancer cells to promote tumor growth is achieved by electrochemical communication at AMPA receptor-dependent neuron-GM synapses [47,48]. The results of these enrichment analyses partially reveal the potential mechanisms by which metal ion stimulation affects GM.

A new approach to disease treatment and prevention called precision medicine takes into account the individual differences in genes, environment, and lifestyle of each person and requires us to accurately categorize diseases on an individual or subgroup basis [49]. Conventional classification of GM patients is based on cell morphology, malignancy, and tumor location, and for classifying them from a precision medicine perspective. We performed consensus clustering of GM patients based on 77 metal ion stimulation-related signature genes. We found 693 GM patients with two distinct response states (subgroups) to metal ion stimulation. Considering the heterogeneity of response states to metal ion stimulation among individuals, we quantified the response to metal ion stimulation in GM patients using tSNE and calculated metal ion stimulation response scores. Metal ion stimulation response scores were significantly higher in M2 than in M1, which suggests potential clinical predictive properties of metal ion stimulation-related genes. In fact, further studies are still needed to investigate how the difference in response scores to metal ion stimulation between M2 and M1 affects GM development and progression. Combined with the clinical characteristics of GM patients, we found that the M2 group accounted for the majority of patients with high-grade GM, and the M1 group had a predominance of patients with IDH mutant phenotypes. Additionally, we found that the immune microenvironment of GM patients responded differently to metal ion stimulation. This, along with the results of functional and pathway enrichment analyses of the differential genes between the M2 and M1 groups, suggests that the variations in the immune microenvironment may be due to stimulation with different metal ions, ultimately affecting neurotransmitter secretion. Previous studies have shown that neurotransmitters have been well-documented to determine the fate of immune cells [[50], [51], [52]]. Our findings seem to support this mechanism and also suggest that the specific role of metal ion stimulation in GM deserves further exploration.

Then, we investigated the value of metal ion stimulation-related feature genes in GM diagnosis and prognosis. We used recursive feature elimination and computation of SHAP values for feature selection on the established XGboot model. The two methods identified 15 overlapping metal ion-related feature genes. Among them, SLC30A3, CRHBP, SYT13, DLG2, CDK1, and WNT5A showed excellent diagnostic value in the external validation set with AUC above 0.90. In addition to the expression changes of SLC30A3 by metal ion stimulation, HDAC1 overexpressed in glioblastoma inhibits the expression of SLC30A3 by deacetylation modification, which is related to the malignant phenotype of glioblastoma [53]. TThe expression of DLG2 affected by AKIP1 is associated with GM proliferation, migration, and invasion [54]. CDK1 has long been shown to be associated with GMgenesis and, the development of GM and has been used as a target for GM therapy [55,56]. WNT5A is affected by lncRNA H19 to promote cell proliferation, migration, and angiogenesis in GM [57]. And overexpression of WNT5A characterizes the most aggressive GMs [58]. In addition, WNT5A expression has been associated with temozolomide resistance [59]. In our prognostic study, CDK1, SLC30A3, and SYT13 were also shown to be biomarkers of prognostic value for GM patients. However, there have been no correlation studies between CRHBP and SYT13 and GM. We hope to explore how they may affect the development and progression of GM through further studies by us or others.

Finally, the role of 14 characteristic genes stimulated by metal ions in distinguishing transgenic levels was analyzed. Among the prediction results of the four models, XGBoost Kmeans showed the most prominent performance in four out of six indicators. But it is the worst among the other two indicators. Considering that its FDR index is also more prominent, we must make corresponding trade-offs based on this result. It is difficult to make trade-offs based solely on single calculation metrics such as sensitivity, specificity, or precision. We delved into the two indicators of FDR and F1 score. These two indicators provide a more comprehensive and comprehensive evaluation of the model. In GM grade prediction, high-grade gliomas are defined as positive labels. A high FDR indicates that many low-level GM patients are mistaken for high-level GM patients. Due to the poorer treatment and prognosis of high-grade GM patients. So it is particularly important to discover as many high-level GM patients as possible. We cannot overly negate a model here just because of its high FDR. The F1 score takes into account both Precision and Recall, aiming to balance the precision and recall of the model. The higher the F1 score, the better the model. FDR and F1 score combined three of our six indicators, and the XGBoost Kmeans model also outperformed in terms of accuracy. Therefore, after comprehensive consideration, the XGBoost Kmeans model is the best predictive model.

In conclusion, this study is the first to identify the subtypes of GM associated with metal ion stimulation. It is also the first study to explore the correlation between metal ion stimulation and the clinical and immunological characteristics of GM. Additionally, this study also improves the prediction performance of the classification model. Based on the DEMISGs of GM patients, different response states (subtypes) to metal ion stimulation in GM patients were identified and validated by consistent clustering. The correlation of GM patients' response states to ionic stimuli with GM grading, IDH status, and immune profile was determined. This enriches our understanding of the complex and subtle relationship between the environment, GM, and the immune system. The DEMISGs were further narrowed down by machine learning to identify metal ion stimulation-related genes that are valuable for the diagnosis and prognosis of GM patients. In the future, we will combine data from other platforms and relevant molecular biology experimental techniques to increase the evidence of existing studies. In-depth study of the relationship between CRHBP SYT13 and GM. We hope to enrich the role of metal ion stimulation in the development of GM and obtain new targets for the treatment of GM in order to provide more accurate and effective treatment for transgenic patients. Furthermore, we will further investigate the application of SHAP values in improving classification prediction performance. We will integrate different tree models to achieve the goal of innovating new algorithms.

## 5 Conclusions

This study provides ample evidence for the extremely important role of metal ion stimulation in the occurrence and development of GM. Additionally, the study also found that SHAP values play a significant role in improving the performance of classification prediction.

## Author contributions

JC designed and drafted the manuscript. JC, JW, ZB,and TW performed data collection and analysis.TW revised important parts of the manuscript. LP and ZB provided guidance for further revision of the manuscript. KY and ZJ provided financial support for this study. All authors have read and approved the manuscript.

## Funding

Our research is in part supported by the National Nature Science Foundation of China (Grant No.20003560), the 10.13039/501100007129Natural Science Foundation of Shandong Province (Grant No. ZR2020MH340), and Undergraduate Education Reform Research Project of Shandong Province(Grant No. M2021174).

## Data availability statement

All data for this study were obtained from publicly available databases and can be accessed at the following URL.

GEO(https://www.ncbi.nlm.nih.gov/geo/).

CGGA(http://www.cgga.org.cn).

TCGA(https://portal.gdc.cancer.gov/).

## Ethics approval and consent to participate

Not applicable.

## Consent for publication

Not applicable.

## Declaration of competing interest

The authors declare that they have no known competing financial interests or personal relationships that could have appeared to influence the work reported in this paper.
